# *Helicobacter pylori* infection rates in dyspeptic Serbian HIV-infected patients compared to HIV-negative controls

**DOI:** 10.1371/journal.pone.0248041

**Published:** 2021-03-10

**Authors:** Aleksandra Radovanovic Spurnic, Zoran Bukumiric, Djordje Jevtovic, Branko Brmbolic, Tatijana Pekmezovic, Dubravka Salemovic, Ivana Pesic Pavlovic, Ivana Milosevic, Jovan Ranin, Milos Korac

**Affiliations:** 1 Clinic for Infectious and Tropical Disease, Clinical Center of Serbia, Faculty of Medicine, University of Belgrade, Belgrade, Serbia; 2 Institute of Medical Statistics and Informatics, Faculty of Medicine, University of Belgrade, Belgrade, Serbia; 3 Institute of Epidemiology, Faculty of Medicine, University of Belgrade, Belgrade, Serbia; 4 Microbiology Department, Clinical Center Serbia, Belgrade, Serbia; Azienda Ospedaliera Universitaria di Perugia, ITALY

## Abstract

*Helicobacter pylori* infection does not belong to the spectrum of opportunistic infections in people living with HIV (PLHIV). To evaluate the *Helicobacter pylori* infection prevalence rate trends in HIV co-infected individuals in comparison to the HIV-negative population, we compared histopathological findings of *H*. *pylori* positive gastritis (gastritis topography and histopathology) between 303 PLHIV and 2642 HIV-negative patients who underwent esophagogastroduodenoscopy (EGD) between 1993 and 2014 due to dyspeptic symptoms. The prevalence of *H*. *pylori* infection was significantly higher in HIV-negative controls than in PLHIV (50.2% vs. 28.1%). A significantly positive linear trend of *H*. *pylori* co-infection in PLHIV was revealed in the observed period (b = 0.030, SE = 0.011, p = 0.013), while this trend was significantly negative in HIV-negative patients (b = - 0.027, SE = 0.003, p < 0.001). Patients with HIV/*H*. *pylori* co-infection had significantly higher CD4^+^ T cell counts and more often had undetectable HIV viremia, due to successful anti-retroviral therapy (ART). Stomach histopathological findings differed between HIV co-infected and *H*. *pylori* mono-infected patients. Our findings confirm that the ART has changed the progression of HIV infection, leading to a significant increase in the prevalence of *H*. *pylori* infection in dyspeptic PLHIV over time. Our data also suggests that a functional immune system may be needed for *H*. *pylori*-induced human gastric mucosa inflammation.

## Introduction

*Helicobacter pylori* (*H*. *pylori*) is the most common bacterial infection in the world [1]. It is estimated that nearly half of the world population harbors this bacteria [2]. Several factors are essential for the infection establishment, and they include environmental, bacterial, and host factors [3, 4].The acute infection is mostly asymptomatic [5]. However, most of the infected individuals cannot overcome the infection, so it may persist for decades, causing chronic gastritis that can sometimes lead to gastric ulcer, or even gastric adenocarcinoma [6] or mucosa-associated lymphoid tissue (MALT) lymphoma, along with various extra-gastrointestinal manifestations of the disease [7].

It has been observed that the prevalence of *H*. *pylori* infection varies over time in different geographical regions, even within the same population. The prevalence is rather low in industrialized countries, while it reaches much higher levels in developing and underdeveloped countries, mostly among children. There are no available data for its prevalence in the Republic of Serbia, while in the neighboring Republic of Croatia it is 60.4% [8].

Human Immunodeficiency Virus (HIV) was isolated and identified as the causative agent of the Acquired Immunodeficiency Syndrome (AIDS) in 1985, at the same time when *H*. *pylori* was discovered [9, 10]. HIV primarily causes the impairment of cell-mediated immunity, which makes the infected individuals highly susceptible to the development of opportunistic infections and opportunistic tumors. It has been shown that *H*. *pylori* does not belong to the spectrum of HIV-related opportunistic infections. *H*. *pylori* may occur in early phases of HIV infection, when the immune function is still relatively preserved [11, 12]. According to the published data, the prevalence of *H*. *pylori* infection among people living with HIV (PLHIV) varies between 10–80% in different geographical regions and pertinent populations [13–17]. We have previously reported that the prevalence of *H*. *pylori* infection among Serbian AIDS patients was 25.8% [18]. Most of the clinical studies aiming to compare the *H*. *pylori* infection in HIV co-infected and in HIV negative patients, demonstrated lower prevalence of this infection among PLHIV than in the HIV negative population. However, the increase of *H*. *pylori* co-infection prevalence was observed in the era of highly active antiretroviral therapy (HAART) [19–21].

The role of HAART and the consequent immune reconstitution, use of antibiotics, as well as proton-pump inhibitors (PPI) and H_2_ blockers, along with hypochlorhydria, body mass index (BMI) and lifestyle on *H*. *pylori* prevalence among PLHIV have all been addressed in numerous studies [11].

The study presented here aimed to analyze the *H*. *pylori* infection incidence among dyspeptic HIV co-infected individuals and HIV negative patients during the period between January 1^st^ 1993 and December 31^st^ 2014, encompassing both the pre-HAART and HAART era.

We also compared histopathological findings of *H*. *pylori-*positive gastritis (gastritis topography and histology) in PLHIV and HIV negative patients.

## Materials and methods

This retrospective case-control study was conducted at the Belgrade University Hospital for Infectious and Tropical Diseases, the reference center for HIV and AIDS management in Serbia.

The Study was approved by the Ethics Committee of Clinical Centre of Serbia and granted by the Ministry of Education, Science and Technological Development of the Republic of Serbia [No. 175073].

The survey protocol was conducted according to the Declaration of Helsinki. All survey participants signed informed consent forms before EGD was performed.

### Study population

#### Study inclusion and exclusion criteria

All patients who had dyspeptic symptoms lasting up to three months before esophagogastroduodenoscopy (EGD) and who underwent their first EGD between January 1993 and December 2014 were included in the study. Dyspeptic symptoms comprised epigastric discomfort (pain), nausea, vomiting, heartburn, burping, body weight loss, bloating and stomach cramps. The study group were PLHIV, while the controls consisted of HIV negative patients from the general population. Patients with gastrointestinal bleeding (haemathemesis, melena), odynophagia, dysphagia (indicating possible opportunistic diseases), chest pain, and those younger than 18 years were excluded from the study.

Each patient/control was enrolled in the study only once.

#### Data collection and instruments

We collected demographic data (age, gender), risk factors for HIV acquisition, duration of HIV, CD4^+^ cell counts, HIV viral load, and the history of antiretroviral therapy (ART) from the hospital database. The CD4^+^ cell counts and HIV viral loads were considered relevant if the blood sample for their determination was taken within 1 month before or after EGD. Histopathological data were collected from the database of the Institute of Pathology, Faculty of Medicine, University of Belgrade.

The HIV status was determined using commercial immunoassays, in accordance with the manufacturer´s protocol. The CD4 cells were quantified by flow cytometry (Becton Dickinson BD FACS Count). Plasma HIV-1 RNA loads were measured with EDTA collected plasma samples, frozen at -20°C, using commercial RT-PCR tests. The usage of PCR tests and their limits of detection, expressed as copies per milliliter (cps/ml), varies over time according to what was available on the market. From 1997 to 2008 Roche Amplicor HIV-1 Monitor Test was used, with the range of detection of 400 cps/ ml– 750000 cps/ml, while from 2008 to 2016 COBAS AmpliPrep/CobasTaqMan HIV version 1.5, with the range of detection from 48 cps/ml– 10 000 000 cps/ml was used, and recently, in 2016, COBAS HIV-1 4800/6800/8800 with the range of detection from 20 cps/ml– 10 000 000 cps/ml of plasma was introduced.

### Endoscopy and histopathology

All patients underwent EGD after the informed consent was signed. EGD was performed in the standard manner using Olympus video endoscope. Four gastric biopsy specimens were taken from each patient (two from the corpus and two from the antrum). Endoscopic diagnosis was recorded using standard terminology. Biopsies were taken from macroscopically normal tissue and macroscopically pathological areas. Biopsy samples were fixed in formalin, embedded in paraffin, cut into 4-μm sections, and stained by hematoxylin and eosin (H&E) and modified Giemsa stain. The stage of gastritis was scored in accordance with the Sydney classification [22].

### *H*. *pylori* diagnosis

Diagnosis of *H*. *pylori* infection was conducted ‎by histopathological examination using a modified Giemsa staining protocol outlined by Gray S et al. [23].‎

### Statistical analyses

All analyses were performed using an electronic database organized in the IBM SPSS Statistics 22 (SPSS Inc., Chicago, IL, USA) statistical package. Patients’ demographic characteristics were analyzed using descriptive statistical methods, while non-parametric variables were analyzed using Fisher’s exact test and Wilcoxon signed-rank test, as appropriate.

While the differences between the groups were analyzed with the chi-square or Fisher`s exact test for the categorical variables, Wilcoxon test was used for the qualitative (2 classes) and numeric variables.

Joinpoint regression program (version 4.3.1) was used to identify trend rates of the prevalence of *H*. *pylori* infection.

The level of significance (alpha level) was set at 0.05.

## Results

We included 2642 patients in this retrospective case-control study. All patients underwent EGD due to various dyspeptic symptoms. In total, 303 HIV-positive patients and 2339 HIV-negative patients were included as cases and controls, respectively.

**Fig 1:**
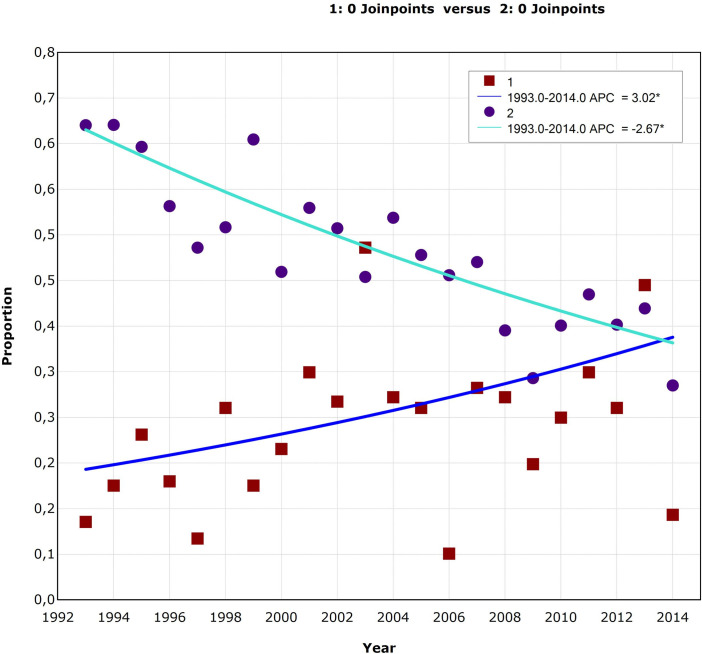
A joinpoint regression analysis of *Helicobacter pylori* prevalence rate trends in HIV-positive (1- red square) and HIV-negative (2- purple circle) patients in Serbia in the period of 1993–2014. Statistically significant trend; APC—annual percent change.

Joinpoint regression analysis revealed that from 1993 to 2014 there was a significant positive linear trend of *H*. *pylori* co-infection among PLHIV (b = 0.030, SE = 0.011, p = 0.013), while the trend of *H*. *pylori* infection in the general population control group was significantly negative (b = - 0.027, SE = 0.003, p < 0.001). Annual percent change (APC) in *H*. *pylori* infection rates in PLHIV was 3.0 (95% CI 0.7 to 5.4), while in controls it was– 2.7 (95% CI –3.3 to –2.0). However, the APC was not significant in either of the two groups (Fig 1).

In the HIV-positive group, there were 221 (72.9%) males and 82 (27.1%) females, with the mean age of 40.2±10.7 years (age range 18–83). *H*. *pylori* infection was diagnosed in 85 (28.1%) participants. PLHIV with and without *H*. *pylori* co-infection were of similar age (p = 0.903). There was no significant difference between sexes (p = 0.565), CD4^+^ T cell counts at the time of HIV diagnosis (p = 0.287), nor the risk factors for HIV infection between the two subgroups (p = 0.512).

At the time of EGD, CD4^+^ T cell counts were shown to be significantly higher in the PLHIV subgroup with *H*. *pylori* co*-*infection, than among those without this co-infection (p<0.001) (Table 1).

**Table 1:** Characteristics of people living with HIV.

Variables	*H*. *pylori* positive	*H*. *pylori* negative	*p-value*
	n = 85	n = 218	
Age (years), mean±SD	40.4±9.9	40.2±11.0	0.903
Gender, *n* (%)			0.565
male	60 (70.6)	161 (73.9)	
female	25 (29.4)	57 (26.1)	
Positive HIV qRT-PCR, n (%)	10 (15.9)	63 (50.8)	**<0.001**
HIV PCR cps/ml, median (range)	0 (0–21000)	20 (0−10^7^)	**<0.001**
CD4^+^ count/μl at the time of HIV diagnosis, median (range)	190 (4–835)	155 (2–1074)	0.287
CD4^+^T cell count/mm^3^ at the time of EGD, median (range)	471 (35–1622)	218.5 (2–1019)	**<0.001**
CD4^+^T cell count/mm^3^ ≥200, n (%)	79 (97.5)	111 (54.4)	**<0.001**
AIDS, n (%)	54 (65.9)	163 (80.7)	**0.008**
ART, n (%)			**0.002**
No therapy	8 (9.8)	59 (29.4)	
Mono/dual	9 (11.0)	14 (7.0)	
HAART	65 (79.3)	128 (63.7)	
*HIV risk behavior*, *n (%)*			0.512
Intravenous drug users	19 (23.2)	51 (25.2)	
Multiple sexual partners	46 (56.1)	117 (57.9)	
Transfusion of blood/blood products	4 (4.9)	14 (6.9)	
Unknown	13 (15.9)	20 (9.9)	

HIV viremia was more frequently undetectable in patients with *H*. *pylori* co-infection, due to successful ART [10 (15.9%) vs. 63 (50.8%) *p <*0.001], while detectable viremia was more common in the subgroup without *H*. *pylori* co-infection [0 (0–21000) cp/ml vs. 20 (0−10^7^) cp/ml (p*<*0,001)]. PLHIV with *H*. *pylori* co-infection had significantly lower prevalence of AIDS (65.9 vs. 80.7%, p = 0.008) (Table 1).

In PLHIV with *H*. *pylori* co-infection, the CD4^+^ T cell counts were significantly higher at the time of EGD compared to the time when HIV infection was detected (p *<*0.001) (Table 2).

**Table 2:** The ratio of CD4^+^ T lymphocyte counts at the time of HIV diagnosis and at the time of esophagogastroduodenoscopy in *H*. *pylori* co-infected patients.

	x;^-^	SD	med	min	max	*p*-value
CD4+T cell count/mm^3^ at the time of HIV diagnosis	218.5	155.7	190	4.0	835.0	**<0.001**
CD4 + count/mm^3^ at the time of EGD	506.8	229.0	471	35.0	1622.0	

In PLHIV with *H*. *pylori* co-infection, the CD4^+^ T cell count was not related to gastritis topography (p = 0.470), *H*. *pylori* density (p = 0.807), gastritis activity (p = 0.333), nor the presence of lymphoid follicles (p = 0.365) and intestinal metaplasia (p = 0.331) (Table 3).

**Table 3:** The influence of CD4^+^ T cell count/mm^3^ values on the histopathological findings in PLHIV with *H*. *pylori* co-infection.

	*n*	*CD4*^*+*^*T cell* count/mm^3^at the time of EGD					*p-value*
		x;^-^	sd	med	min	max	
**Gastritis topographical localization, n (%)**							0.470
Antrum	31	493.8	290.6	440.0	35.0	1622.0	
Corpus	4	493.5	72.9	467.5	440.0	599.0	
Pan-gastritis	46	516.8	190.7	499.5	172.0	1046.0	
***H*. *pylori* density, n (%)**							0.807
Mild (+)	28	482.0	175.8	450.0	230.0	869.0	
Moderate (++)	27	553.9	316.5	499.0	35.0	1622.0	
Severe (+++)	26	484.9	163.5	473.5	172.0	1005.0	
**Activity, n (%)**							0.333
No	2	348.5	72.8	348.5	297.0	400.0	
Mild (+)	48	507.3	256.9	452.5	35.0	1622.0	
Moderate (++)	24	523.6	161.1	551.0	172.0	772.0	
Severe (+++)	6	526.8	276.0	473.5	210.0	1005.0	
**Lymphoid follicles, n (%)**							0.365
No	51	522.2	243.8	476.0	230.0	1622.0	
Mild (+)	23	470.9	213.4	450.0	35.0	1046.0	
Moderate (++)	5	580.0	153.3	596.0	334.0	757.0	
**Intestinal metaplasia, n (%)**							0.331
No	66	516.4	226.1	478.0	172.0	1622.0	
Mild (+)	13	432.1	187.3	416.0	35.0	772.0	

The control group consisted of 2339 HIV-negative patients from the general population. Their gender ratio was 1012 (43.3%) males vs. 1327 (56.7%) females, with a mean age of 46.3±14.7 years (age range 18–86). *H*. *pylori* infection was detected in 1174 (50.2%) participants. *H*. *pylori-*infected subjects were significantly older than those without this infection (47.5±13.6 vs. 45.0±15.5 years, *p<*0.001). *H*. *pylori* infection did not show any gender-related correlation (p = 0.241) (Table 4).

**Table 4:** Characteristics of HIV-negative patients.

Variables	*H*. *pylori* positive	*H*. *pylori* negative	p-value
	n = 1174	n = 1165	
Age (years), mean±SD	47.5±13.6	45.0±15.5	**<0.001**
Male *n* (%)	522 (44.5)	490 (42.1)	0.241
Female ***n* (%)**	652 (55.5)	675 (57.9)	

*H*. *pylori* infection was significantly more prevalent in the HIV-negative group of patients in comparison to PLHIV (50.2% vs. 28.1%). The control group of patients with *H*. *pylori* infection was significantly older than the PLHIV group with *H*. *pylori* co-infection (47.5±13.6 vs. 40.9±9.9; p≤0.001). The histopathological grading of gastritis activity differed significantly between those two subgroups (p = 0.006). The mildest cases prevailed in PLHIV, while in the control group there were more patients with moderate and severe activity. There was no difference between two *H*. *pylori* positive subgroups in gastritis topographical localization (p = 0.452), *H*. *pylori* density (p = 0.905), presence of lymphoid follicles (p = 0.074) nor intestinal metaplasia (p = 0.817) (Table 5).

**Table 5:** Differences in histopathological findings in PLHIV and HIV-negative patients with *H*. *pylori* infections.

	HIV positive	HIV negative	*p value*
	*H*. *pylori* positive	*H*. *pylori* positive	
n (%)	85 (28.1)	1174 (50.2)	**<0.001**
**Gastritis topographical localization, n (%)**			0.452
Antrum	32 (37.6)	466 (39.9)	
Corpus	4 (4.7)	29 (2.5)	
Pan-gastritis	49 (57.6)	673 (57.6)	
***H*. *pylori* density, n (%)**			0.905
Mild (+)	29 (34.1)	384 (32.8)	
Moderate (++)	29 (34.1)	444(37.9)	
Severe (+++)	27 (31.8)	343 (29.3)	
**Activity, n (%)**			**0.006**
No	3 (3.5)	27 (2.3)	
Mild (+)	49 (58.3)	516 (44.1)	
Moderate (++)	25 (29.8)	466 (39.9)	
Severe (+++)	7 (8.3)	160 (13.7)	
**Lymphoid follicles, n (%)**			0.074
No	54 (65.1)	634 (55.0)	
Mild (+)	24 (28.9)	418 (36.3)	
Moderate (++)	5 (6.0)	100 (8.7)	
**Intestinal metaplasia, n (%)**			0.817
No	69 (83.1)	978 (84.3)	
Mild (+)	14 (16.9)	167 (14.4)	
Moderate (++)	0 (0)	15 (1.3)	

## Discussion

Considering the already published research data on *H*. *pylori* infection among PLHIV, this is the first report in which the joinpoint regression analysis was performed. Also, to the best of our knowledge, this is the first EGD-based morphological comparison of histopathological findings in *H*. *pylori-*positive patients with and without HIV co-infection covering 21 years.

Our data suggest that the prevalence of *H*. *pylori* infection is significantly lower among PLHIV in comparison with the HIV-negative population, over 21 years covered by our study. These data are in concordance with those already published addressing this issue [11, 12, 14, 15, 17, 19–21]. In addition, we found that the prevalence rates of *H*. *pylori* infection during more than 20 years of EGD follow-up among patients with dyspeptic symptoms, had opposed linear trends between the two investigated groups. Decreasing prevalence rates of *H*. *pylori* infection over time were observed in the HIV-negative population, contrary to the increasing trend in PLHIV. Moreover, the APC increase was 3% in the PLHIV group while in the general population the APC decrease was 2.7%. Even though we did not demonstrate any significant changes in two APCs, which converge towards similar prevalence rates during last two years of the observed period, we may speculate that these changes in the two observed linear trends could be related to the gradual ART introduction in HIV-positive patients, as well as the eradication therapy for *H*. *pylori* among controls.

The first patient with AIDS in Serbia was diagnosed in 1985, while ART, including mono and dual therapy, became available in 1987 and was in use up to 1998, when HAART became widely available [24]. We demonstrated that HAART reduces HIV-related morbidity and mortality, both in men and women, as shown in other observational studies in developed and developing countries, where this treatment is available [25]. By providing durable viral suppression, HAART corrects many of the immune defects caused by HIV, including restoration of opportunistic pathogen-specific immune responses and subsequent regression or prevention of opportunistic infections [26, 27]. Immune reconstitution may allow for inflammatory response-related unmasking of some previously silent infections, the phenomenon called Immune Restoration Inflammatory Syndrome (IRIS) [28, 29].

Standard and highly efficient triple *H*. *pylori* eradication therapy has been available in Serbia since it had been advocated by most guidelines, and this treatment approach has led to the decreased shedding of the pathogen, along with the decreased prevalence of the infection in the general population [30]. This epidemiological phenomenon is rather different than what has been observed among PLHIV during both the pre-HAART and HAART eras. The rising linear trend of *H*. *pylori* infection in the PLHIV population over time could probably be explained by the less effective mono and dual ART in comparison to HAART with respect to immune reconstitution, which we consider to be important for the pathogenesis of *H*. *pylori*-induced gastric mucosa inflammation.

It seems that the most important factor related to the occurrence of *H*. *pylori* infection in PLHIV is the immune restoration, corresponding to the CD4^+^ T cell count. In this study we showed that there was no difference in the CD4^+^ T cells counts between PLHIV with and without *H*. *pylori* co-infection at the time of HIV diagnosis. Contrary to this, at the time when EGD was performed, PLHIV with *H*. *pylori* co-infection had a significantly higher CD4^+^ T cell counts, mostly over 200/mm^3^, and less progression to AIDS. However, once a higher CD4^+^ T cell count is achieved, suggestive of immune reconstitution, it does not affect the histopathological findings of the gastric mucosa. The topographic localization of gastritis, *H*. *pylori* density, activity, the presence of lymphoid follicles and intestinal metaplasia in patients with HIV/*H*. *pylori* co-infection was not dependent on the level of immune restoration, monitored by the CD4^+^ T cell counts. Definitely T cell subpopulations affect *H*. *pylori* colonization, but there is not yet sufficient evidence that T cell deficiency causes *H*. *pylori* removal.

When we compared histopathological findings between HIV-positive and HIV-negative subjects with *H*. *pylori* infection, we only found the difference in activity: most of the PLHIV had mild gastritis activity (58%) while HIV-negative patients mostly had moderate/severe gastritis activity (53.6%), which may be attributed to the incomplete immune recovery in co-infected patients. However, in our previous investigation, where we enrolled 212 HIV/*H*. *pylori* co-infected patients, we concluded that most of the patients treated in the pre-HAART era had mild gastritis activity while those on HAART mostly had mild/moderate gastritis activity [21].

In this retrospective study we did not evaluate if the usage of antibiotics affected the prevalence of *H*. *pylori* infection over time. Antibiotics are used for *H*. *pylori* eradication in both PLHIV and HIV-negative patients. *H*. *pylori* eradication therapy consists of simultaneous usage of two antibiotics from different classes along with proton pump inhibitor [31], and antibiotic monotherapy had only minor efficacy in *H*. *pylori* eradication [32].

In addition, some PLHIV in our cohort had been taking antibiotics earlier to treat opportunistic infections, such as *Mycobacterium tuberculosis* infection. Rifampicin has an excellent *in vitro* and good efficacy *in vivo* against *H. pylori [33]*, but does not necessarily result in eradication of *H*. *pylori* infection [34]. While we cannot rule out clearance of H. pylori infection in some of our PLHIV patients previously treated for tuberculosis, the available published studies do not provide a definitive answer on the effect of rifampicin use on the incidence of *H*. *pylori* infection in PLHIV treated for tuberculosis. Their findings, however, suggest that progressive HIV disease rather than antibiotic usage may be responsible for the diminution in frequency of *H*. *pylori* co-infection [35]. We can speculate that in the era of less effective antiretroviral therapy, when tuberculosis incidence in PLHIV was higher, the use of rifampicin might have contributed to the lower incidence of *H*. *pylori* infection in PLHIV more significantly than in the HAART era. Still, we cannot exclude immunodeficiency of these patients as an important contributing factor to their lower incidence of *H*. *pylori* infection. It is important to note that not all of the patients included in this study had been treated for tuberculosis. Future research is needed to clarify the impact of previous antibiotic therapy, primarily antituberculosis therapy, on the incidence of *H*. *pylori* infection in PLHIV. Co-trimoxazole has not been reported to have activity against *H*. *pylori* [36].

Since *H*. *pylori* induced gastric inflammation is less severe in PLHIV, it is possible that *H*. *pylori* infection in these patients was underdiagnosed due to mild symptoms, especially before HAART introduction. In addition, dyspeptic symptoms are common in PLHIV and are usually not associated with *H*. *pylori* as they may be due to opportunistic infections and/or tumors, diet, ART side effects, etc., with peptic ulcer disease and gastroesophageal reflux disease frequently detected by EGD [18, 37].

Our previous studies demonstrated that the prevalence of *H*. *pylori* gastritis was higher among PLHIV with less advanced HIV infection and hence more preserved immune function. We previously demonstrated that the prevalence of *H*. *pylori* gastritis had been increasing in concert with the HAART efficacy evolution and the consequent improved immune reconstitution [21]. More research is needed to elucidate whether immune function, rather than some local factors influence the prevalence of *H*. *pylori* gastritis among PLHIV.

This is a retrospective case-control study and therefore some potentially relevant information was not available, such as other possible risk factors for HIV/*H*. *pylori* co-infection in addition to the ART, e.g. exposure to antibiotics, PPI and H_2_ blocker therapy, body mass index, etc. Also, we did not explore the *H*. *pylori* acquisition in the control, HIV-negative group in detail.

Despite these limitations we believe that the study presents new research findings on the histopathology of *H*. *pylori* positive gastritis in HIV/*H*. *pylori* co-infection and annual percentage changes in *H*. *pylori* infection rates.

## Conclusions

In conclusion, according to our data, we believe that in the population of HIV infected individuals the linear trend of the slowly rising prevalence of gastritis associated with *H*. *pylori* co-infection could be the consequence of slow and steady immune reconstitution ranging from a rather weak mono or dual ART in the beginning to the more potent modern HAART, resulting in a similar level of *H*. *pylori* gastritis development in all our patients.

Our research has shown that *H*. *pylori* infection is present only in PLHIV who have preserved CD4 counts, hence, immunity. Although more research is needed in this area, we believe that our conclusions are extremely important for the longer life expectancy of PLHIV as well as the carcinogenicity of *H*. *pylori*. We believe that the future outcome and course of HIV infection will be quite different and that diseases not previously studied in the context of HIV will become as common in PLHIV as in HIV-negative patients. No studies conducted after HAART became available have implicated that PLHIV individuals are at a decreased risk of gastric cancer. Therefore, PLHIV with *H*. *pylori* co-infection, regardless of their CD4 status, could be at an equal risk for of gastric cancer as HIV negative patients, but less likely to be diagnosed with *H*. *pylori* due to reduced gastric inflammation. Testing for and treatment of *H*. *pylori* in the HIV positive population should be as aggressive as it is for the HIV negative population.

To definitively determine the risk factors for HIV/*H*. *pylori* co-infection, future prospective studies are needed to address the epidemiological characteristics of *H*. *pylori* infection in HIV positive population.

## Supporting information

S1 Text Data for Fig 1. A joinpoint regression analysis of *Helicobacter pylori* prevalence rate trends in HIV-positive (1) and HIV-negative (2) patients in Serbia in the period of 1993–2014.
(TXT)

S1 Table (XLSX)

S2 Table (XLSX)

S3 Table (XLSX)

S4 Table (XLSX)

S5 Table (XLSX)
